# Deauville Score-Based Evaluation of Interim PET/CT in Follicular Lymphoma: A Prognostic Factor Systematic Review and Meta-Analysis

**DOI:** 10.7759/cureus.75169

**Published:** 2024-12-05

**Authors:** João M Fonseca, Sanda K Zloic, Chukwudi I Ayogu, Karabo K Marole, Gianluca C Ingold, Sarah V Moreira, Marco A Soato Ratti

**Affiliations:** 1 Radiology, Hospital Geral de Salvador, Salvador, BRA; 2 Radiology, Special Hospital Agram, Zagreb, HRV; 3 Obstetrics and Gynaecology, Royal Liverpool University Hospital, Liverpool, GBR; 4 Medicine and Surgery, St. George's University School of Medicine, St. George, GRD; 5 Radiology, Hospital Universitario Austral, Pilar, ARG; 6 Radiology, Hospital Universitário da Universidade Federal de Juiz de Fora, Juiz de Fora, BRA; 7 Musculoskeletal Radiology, Fleury Group, São Paulo, BRA

**Keywords:** deauville score, follicular lymphoma, interim petct, prognostic factor, progression free survival

## Abstract

Follicular lymphoma (FL) is an indolent non-Hodgkin lymphoma subtype, posing challenges in prognostication. While interim PET/CT is a recognized response assessment tool in other lymphoma subtypes, its prognostic value for FL remains uncertain. This study aims to evaluate the significance of interim PET results, which were assessed using the Deauville Score. A systematic review and meta-analysis were conducted across several databases, including studies that evaluated interim PET during frontline chemoimmunotherapy, with outcomes adjusted for other prognostic factors. Data extraction and risk of bias were performed independently by two authors. Hazard ratios (HR) and 95% confidence intervals (CI) were pooled using random-effects models, with leave-one-out sensitivity analyses to further assess the results. Four studies involving 427 patients were included. The pooled HR for Progression-Free Survival (PFS) was 2.88 (95% CI: 1.83-4.52; I² = 55.5%; *p *<0.0001), indicating a significant association between positive PET results and poorer PFS. The pooled HR for overall survival (OS) was 3.32 (95% CI: 1.34-8.23; I² = 74.4%; *p *= 0.0097), reflecting a significant decrease in OS with positive PET results. Sensitivity analyses confirmed the significance of these findings. Thus, positive interim PET results are associated with worse outcomes in frontline-treated FL patients, suggesting their potential as a valuable prognostic tool.

## Introduction and background

Follicular lymphoma (FL) is a common and indolent, non-Hodgkin lymphoma (NHL) subtype with an incidence rate of 3.5 cases per 100,000 in the United States from 2000 to 2016 [1]. Current guidelines recommend initial staging using positron emission tomography/computed tomography (PET/CT) to improve accuracy and response assessment if radiotherapy is planned [2]. First-line treatment is guided by staging and therapeutic goals, but the combination of the anti-CD20 monoclonal antibody Rituximab and chemotherapy is the commonly preferred frontline regimen [3-5].

Response evaluation performed with PET/CT is well established at the end of induction of chemoimmunotherapy (CIT) as persistency of positive results indicates a worse prognosis [6]. The Deauville 5-point scoring system (DS) is the currently accepted standardized method for qualitative response assessment in FL with PET/CT [7]. However, patients are typically assessed only after completing the full course of CIT, as there is no established guideline evidence level recommending the use of interim PET/CT for response assessment in FL patients [2]. In contrast, the prognostic impact of interim PET/CT is better established in other lymphoma subtypes such as diffuse large B-cell lymphoma (DLBCL) and advanced-stage Hodgkin lymphoma (HL) [8,9].

Multiple studies have evaluated the results of interim PET/CT in FL patients’ disease progression, showing discrepant results [10-12]. Although most of them lack adjustment for other prognostic factors or fail to specify the method used for PET/CT categorization, a tendency towards a worse prognosis in patients with a positive exam is noticed. In contrast, a prior systematic review was performed in 2016 [13] and reported a lack of supporting evidence for interim PET/CT use in follicular lymphoma but did not limit inclusion criteria for DS-based assessment and adjusted prognostic factor studies.

The aim of this systematic review and meta-analysis is to evaluate the multivariate-adjusted prognostic value of interim PET/CT results, assessed with the Deauville Score, in FL patients during frontline CIT, with the goal of determining its potential as a tool for earlier treatment adjustments and better patient prognosis communication.

## Review

Materials and Methods

Registration and Protocol

This systematic review and meta-analysis were performed and reported in accordance with the Cochrane Collaboration guide to systematic review and meta-analysis of prognostic factor studies and Preferred Reporting Items for Systematic Reviews and Meta-Analysis (PRISMA) Statement Guidelines [14]. Additionally, we registered the study with PROSPERO prior to the initial literature search (CRD42024545907).

Eligibility Criteria and Endpoints

We restricted inclusion to manuscripts and conference abstracts that met the following criteria: (1) randomized trials or nonrandomized cohorts that involved patients diagnosed with follicular lymphoma who underwent frontline CIT; (2) had an interim PET/CT exam evaluated using the Deauville 5-Point Score within 2-5 cycles of treatment; and (3) reported one of the outcomes of interest adjusted to other prognostic factors with hazard ratios (HR). We excluded studies if (1) they did not specify the use of DS for PET/CT evaluation; (2) they involved overlapping populations or lacked distinction between follicular lymphoma and other NHL patients; (3) they included patients previously treated with chemoimmunotherapy or with relapsed/refractory follicular lymphoma; (4) did not apply multivariate analysis to adjust for other prognostic factors; or (5) deliberately changed treatment based on interim PET results. Outcomes included progression-free survival (PFS) and overall survival (OS).

Search Strategy and Data Extraction

A systematic literature search was performed across multiple databases, including MEDLINE, Embase, and the Cochrane Library, from inception to May 2024. The search strategy included terms related to "follicular lymphoma," "PET-CT," "18F-FDG PET/CT," and "interim positron emission tomography." Data extraction was independently performed by two authors (J.F. and S.K.) following predefined search criteria and quality assessment. Discrepancies between reviewers were resolved through discussion or third-party adjudication. Data collected included study characteristics, patient demographics, treatment regimens, PET/CT evaluation methods, follow-up periods, and adjusted prognostic factors. 

Quality Assessment and Risk of Bias

The quality of the included studies was assessed using the QUIPS (Quality In Prognosis Studies) tool as recommended by Cochrane’s Guide to Systematic Review and Meta-Analysis of Prognostic Studies [14]. The tool evaluates studies across six domains: study participation, study attrition, prognostic factor measurement, outcome measurement, study confounding, and statistical analysis and reporting. Two independent authors completed the assessment. Disagreements were solved through consensus and third-party adjudication when necessary. Given the small number of included studies, publication bias analysis was not performed outside of sensitivity analysis. 

Statistical Analysis

Meta-analyses were conducted using both fixed-effect and random-effects models, applying the inverse variance method to pool hazard ratios and their 95% confidence intervals (CIs). Heterogeneity was assessed using the I² statistic, with significance tested using Cochran’s Q test, p-values < 0.10 and I² > 25% were considered significant for heterogeneity. The random-effects model was preferred when substantial heterogeneity (I² > 50%) was detected. For studies missing confidence intervals (CIs), these were estimated using the reported HR and sample size, applying standard formulas for standard error (SE) and CI calculation. Leave-one-out sensitivity analyses were performed by excluding individual studies to assess the robustness of the results and account for the effect of different PET/CT thresholds applied by the studies. We used RStudio 2024.04.2+764 for the required analysis.

Results

Study Selection and Baseline Characteristics

**Figure 1:**
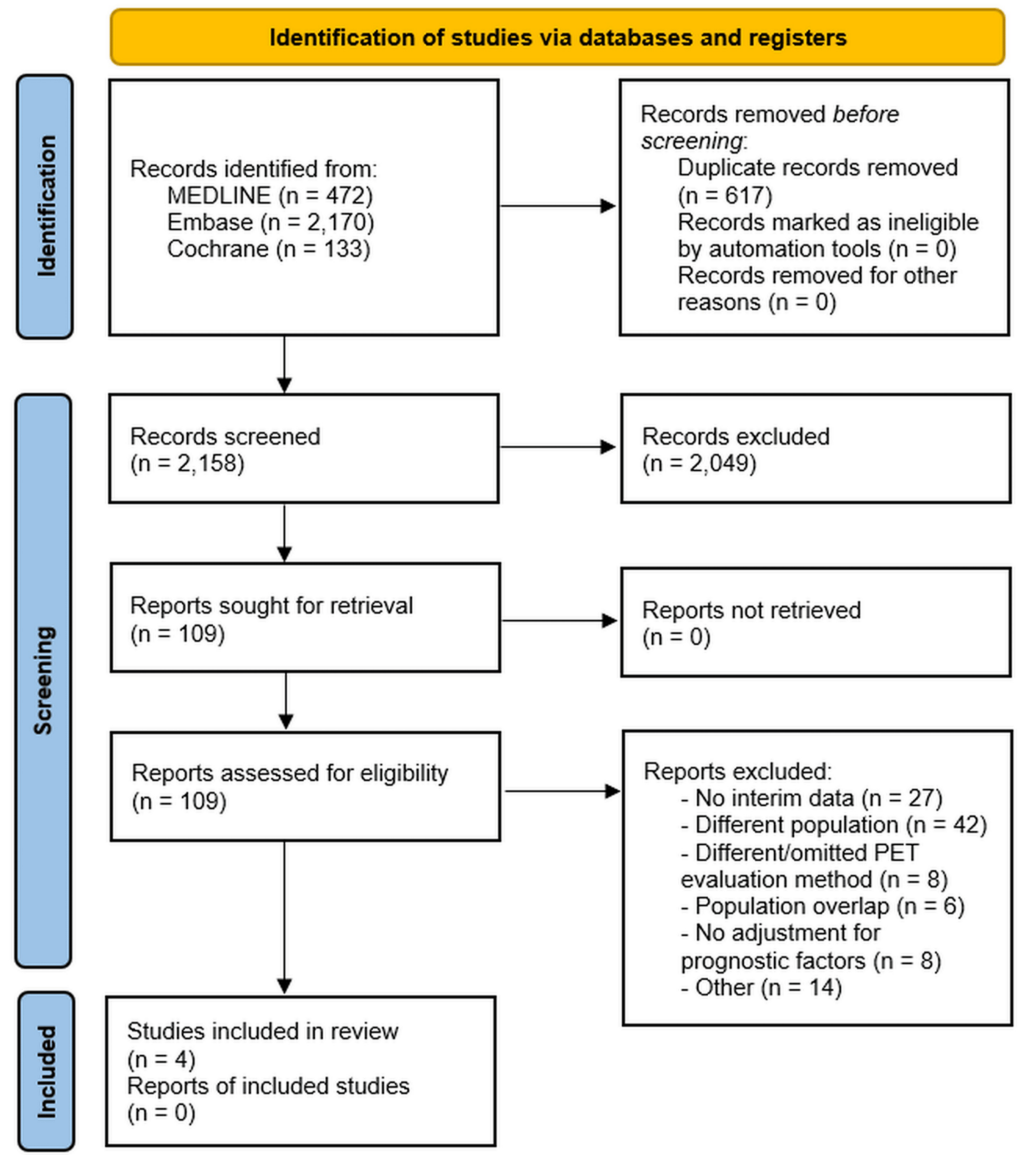
PRISMA 2020 flow diagram of the study

The initial search yielded 2775 records. After duplicate removal, 617 records were excluded. After screening the titles and abstracts of the remaining 2,158 studies, 109 full-text articles were assessed for eligibility. Ultimately, 4 studies comprising a total of 427 patients met the inclusion criteria and were included in the final analysis (Figure 1) [15-18].

The weighted mean age of the population from the included studies was 59.1 years. Approximately 38% of patients had a high Follicular Lymphoma International Prognostic Index (FLIPI) score, and most of the patients had advanced-stage lymphoma. The most commonly prognostic factor adjusted for was the FLIPI score and the CIT regimen. Three studies set the threshold for a positive DS result as 4-5, and one considered the result positive as 3-5 based on the observed criteria (Table 1).

**Table 1:** Baseline characteristics of included studies §: conference abstracts; †: mean or median; PFS: Progression-Free-Survival; OS: Overall Survival; DS: Deauville 5-point Score; PET: Positron Emission Tomography; CIT:  Chemoimmunotherapy; RCT: Randomized Clinical Trial; RCHOP: Rituximab + Cyclophosphamide + Hydroxydaunorubicin + Vincristine (Oncovin) + Prednisone; BR: Rituximab + Bendamustine; FLIPI: Follicular Lymphoma International Prognostic Index; SUVmax: Maximum Standardized Uptake Value; NA: Not Available.

Study	Patients	Age^†^ (years)	Female %	CIT Regimen (RCHOP/BR/Other) %	Stage (Advanced %)	DS Cutoff	PET Timing (Cycles)	Rituximab Maintenance %	FLIPI Score (High %)	Follow-up^†^, mo	Adjusted Prognostic Factors	PFS Definition	OS Definition
Merryman 2023	128	55 (27-83)	48	50/44/6	84	1-2/3-5	2-4	31	32	59	FLIPI; CIT Regimen; Bulk; Age; Sex; Grade; Baseline SUVmax; R Maintenance	Time from initiation of CIT to death from any cause, relapse, or progression, with patients censored at the last time seen alive and progression-free.	Time from initiation of CIT to death from any cause, with patients censored at the last time seen alive.
Durmo 2022^§^	123	60	NA	NA/44/NA	NA	1-3/4-5	4-5	NA	32	NA	FLIPI; CIT Regimen	NA	NA
Laverdure 2022^§^	143	64 (28-86)	NA	15/17/68	88	1-3/4-5	2-4	59	46	67	FLIPI, Histological Grade at Diagnosis	NA	NA
Boo 2019	33	50 (29-84)	48.5	21/0/12	93.9	1-3/4-5	3-4	81.8	48.5	41.6	NA	Period from diagnosis to the disease progression or the last follow-up date.	NA

Quality Assessment

In the risk of bias analysis, two of the studies were assessed to have a low risk of bias (Merryman and Boo) [16,18], with most domains rated as low risk and only one of the domains rated as moderate or high risk, respectively. The other two studies were classified as having a moderate risk of bias (Durmo and Laverdure) [15,17], with multiple domains showing moderate to high risk, particularly in the domains of study participation and confounding (Table 2). 

**Table 2:** Risk of bias of included studies evaluated in the six domains of the QUIPS (Quality In Prognostic Studies) tool summary

Study	Study Participation	Study Attrition	Prognostic Factor Measurement	Outcome Measurement	Study Confounding	Statistical Analysis and Reporting
Merryman 2023	Low	Low	Moderate	Low	Low	Low
Durmo 2022	Moderate	Moderate	Low	Moderate	High	Moderate
Laverdure 2022	Moderate	Low	Moderate	Moderate	Moderate	Moderate
Boo 2019	Low	Low	Low	Low	High	Low

Progression-Free Survival Analysis

**Figure 2:**
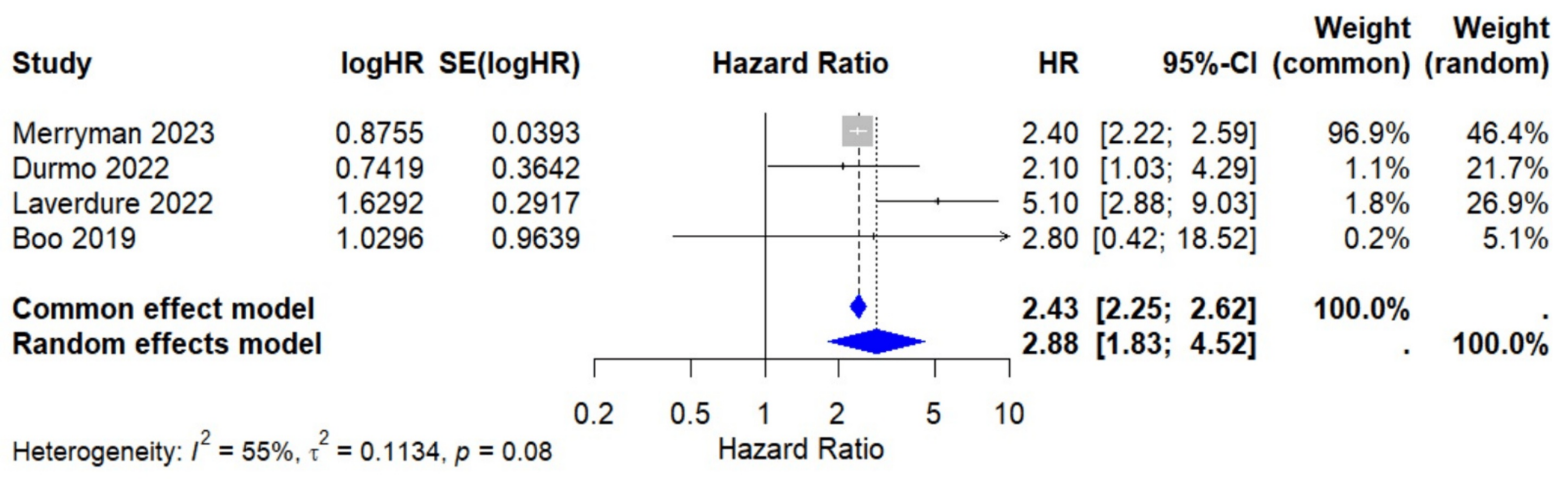
Forest plot for interim PET/CT on progression-free survival HR: Hazard ratio; SE: Standard error; CI: Confidence interval

**Figure 3:**
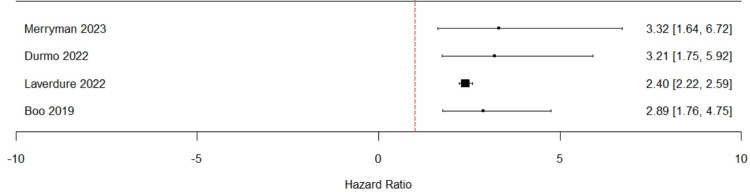
Forest plot for leave-one-out sensitivity analysis for progression-free survival The red dotted line represents Hazard ratio = 1. The study name represents the pooled Hazard ratio excluding the respective study.

The meta-analysis included four studies that reported multivariate analysis of PFS. In the Merryman [18] study, which did not provide confidence intervals, these were approximated using the reported HR and sample size. The pooled HR for PFS was 2.88 (95% CI: 1.83-4.52; I² = 55.5%; p <0.0001) under the random-effects model, indicating a significant association between positive interim PET/CT results and worse progression-free-survival (Figure 2). Moderate heterogeneity was observed (I² = 55.5%; p = 0.08). Leave-one-out sensitivity analysis excluding individual studies showed that the results were robust, with the overall HR remaining significant (Figure 3). The exclusion of the Laverdure [17] study reduced heterogeneity to 0%, indicating this study was likely the primary source of variability. Excluding Merryman [18] from the results caused an increase in the pooled HR and a decrease in heterogeneity: 3.32 (95% CI: 1.64-6.72; I² = 45.7%), suggesting this study had a key role in stabilizing the overall estimate. 

Overall Survival Analysis

**Figure 4:**
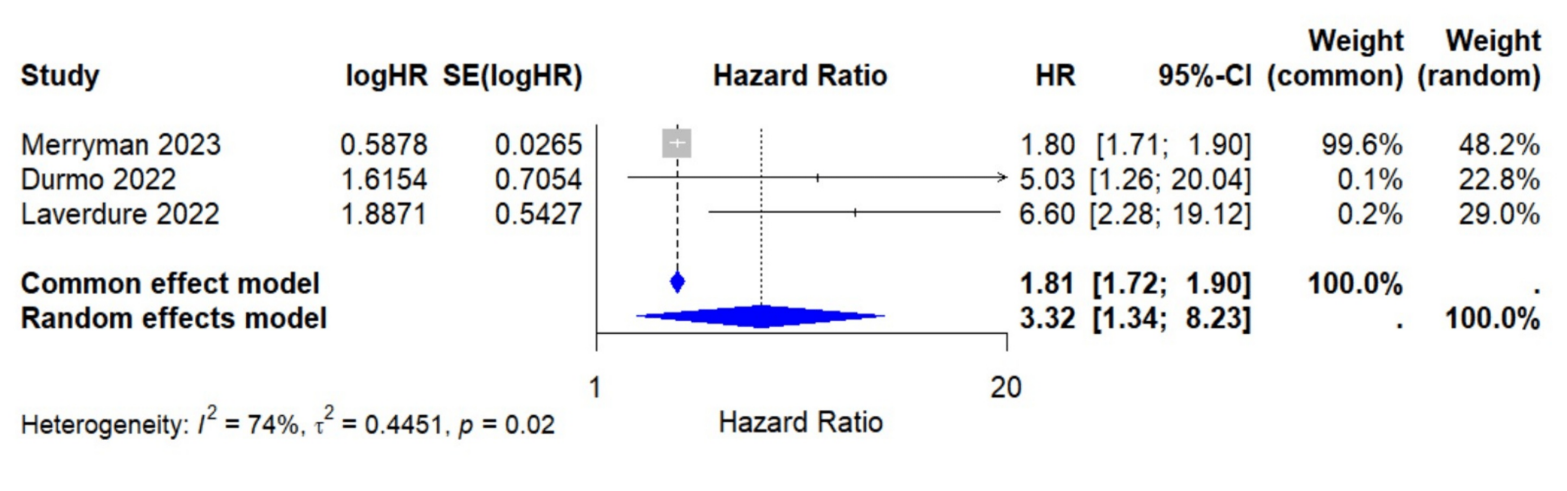
Forest plot for interim PET/CT result on overall survival HR: Hazard ratio; SE: Standard error; CI: Confidence interval

**Figure 5:**
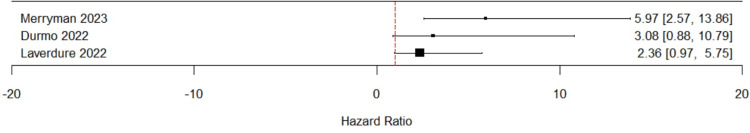
Forest plot for leave-one-out sensitivity analysis for overall survival The red dotted line represents Hazard ratio = 1. The study name represents the pooled Hazard ratio excluding the respective study.

For OS, only three of the included studies reported extractable results for the random-effects model analysis that yielded a pooled HR of 3.32 (95% CI: 1.34-8.23; I² = 74.4%; p = 0.0097), indicating a significant decrease in overall survival associated with positive interim PET results (Figure 4). Substantial heterogeneity was observed (I² = 74.4%; p = 0.02). Excluding Merryman [18] resulted in a higher pooled HR: 5.97 (95% CI: 2.57 - 13.86; I² = 0%; p <0.0001) and eliminated heterogeneity. Excluding Durmo [15] increases heterogeneity and causes the random-effects model to lose significance: HR = 3.08 (95% CI: 0.88 - 10.79; I² = 82.5%; p = 0.0788), suggesting that the study helps reduce variability. Excluding Laverdure [17] reduced heterogeneity but still left moderate variability, and the pooled HR approached significance under the random-effects model: HR = 2.36 (95% CI: 0.97 - 5.75; I²: 52.8%; p = 0.0579) (Figure 5). 

Discussion

To our knowledge, this is the first systematic review and meta-analysis evaluating interim PET/CT results using the Deauville Score as a prognostic factor in patients with follicular lymphoma during frontline CIT. In this analysis of four studies and 427 patients, the main findings included: (1) a significantly worse prognosis in regards to PFS in patients with a positive interim PET result in comparison to those with a negative result; (2) a significant decrease in OS associated with patients that had a positive interim result compared to the patients that did not.

These findings are similar to the results of other studies that applied an unadjusted analysis of interim PET/CT or used criteria other than the DS for exam evaluation [10-12,19-22]. The results are also compatible with those found in previous meta-analyses evaluating the prognostic value of interim PET/CT in patients with different lymphoma subtypes such as HL and DLBCL [23,24], despite having a lower number of included studies and patients.

In order to include all the available studies that reported a multivariate-adjusted analysis, we opted to include the Merryman [18] study despite the difference in DS positivity threshold knowing it would likely contribute to an increase in heterogeneity and decrease separation of PFS and OS curves [25]. However, due to the fact that the study opted for a lower threshold, considering DS 3-5 as a positive result, it favors a more conservative interpretation approach, steering the results toward a lower HR and significance value, as was observed in the leave-one-out sensitivity analysis performed. The pooled HR, however, remained significant in the included study.

Merryman [18] also included a separate analysis for a high-grade FL cohort in their study. Although not part of the exclusion criteria of this review, we opted to not include these results as it would likely increase bias substantially, and only 1% of the participants included were graded as 3B. This decision was also based on the conflicting results of studies evaluating disease progression between grades 1-3A, grade 3B, and DLBCL [26-28].

A high FLIPI score was observed in over one-third of the included population of the study and was the confounding prognostic factor most included in the multivariate analysis of the primary studies (Table 1). These results are in accordance with previous studies that highlight the FLIPI score as a significant prognostic predictor for PFS, OS, and histological transformation in both de novo and relapsed FL [29-31].

There were also reports of seven patients who changed therapy based on the results of interim PET/CT across the included studies. We considered this value acceptable within the limitations of our study.

Earlier treatment adjustment based on response assessment is, however, one of the goals of this systematic review and meta-analysis. Tailored treatment regimens, including chimeric antigen receptor T (CAR-T) cell therapy and rituximab maintenance, could be interesting alternatives to be explored in future trials for high-risk interim PET-positive FL patients, as similar trends are already explored for HL with mixed results [9,32]. 

One of the limitations of our study, as occurs in most prognostic factor studies, is that it is subject to important bias due to other unadjusted prognostic factors. Therefore, the decision to only include primary studies that performed an analysis adjusted to the main prognostic confounders, per Cochrane recommendations [14], resulted in a small number of included studies and participants. There is also the issue of a different PET/CT result threshold in one of the included studies (Merryman) [18] and the moderate to high heterogeneity found. To address these factors, a leave-one-out sensitivity analysis was performed, yielding similar results in the final pooled results. As we included both manuscripts and conference abstracts, the lack of a clear definition of the adjusted prognostic confounders in one of the included studies and discrepancies in the definition of the outcomes is, possibly, another source of bias.

## Conclusions

In conclusion, the results of this systematic review and meta-analysis highlight the significant prognostic value of interim PET/CT, assessed using the Deauville Score, for predicting both progression-free survival and overall survival in patients with follicular lymphoma. Positive interim PET results were strongly associated with worse outcomes, supporting its potential as a valuable tool for early prognostic risk stratification. While the findings are promising, the small number of included studies and moderate to high heterogeneity limits the generalizability of the results. Increased quantity and improved quality data from primary studies are still lacking and are essential to confirm the role of interim PET/CT in clinical decision-making for follicular lymphoma patients.
